# Nature Connectedness and Work Engagement in the Relationship Between Emotional Exhaustion and Life Satisfaction Among Employees

**DOI:** 10.3390/ejihpe16060079

**Published:** 2026-06-05

**Authors:** Soraya Jaime-Jorge, Cristina García-Ael, Garazi Azanza, Ana María Fernández-Fernández, Gabriela Topa

**Affiliations:** 1 Department of Social and Organizational Psychology, Faculty of Psychology, Universidad Nacional de Educación a Distancia (UNED), 28040 Madrid, Spain; cgarciaael@psi.uned.es (C.G.-A.); afernande1312@alumno.uned.es (A.M.F.-F.); gtopa@psi.uned.es (G.T.); 2 Faculty of Social and Human Sciences, University of Deusto, 48007 Bilbao, Spain; garazi.azanza@deusto.es

**Keywords:** work stress, emotional exhaustion, life satisfaction, connectedness to nature, work engagement

## Abstract

This paper analyses associations between psychological health at work and life satisfaction among employees. Specifically, it investigates the association between work stress and emotional exhaustion and explores whether connectedness to nature and work engagement are involved in the indirect association between emotional exhaustion and life satisfaction. Structural Equation Modelling (SEM) was used to analyse data from a final sample of 1851 Spanish workers from diverse professional categories. These included technical and support professionals, administrative employees, middle managers, civil servants and healthcare professionals, unskilled workers, and other occupational groups. SEM analyses were conducted with 1850 valid cases due to one missing or invalid case in the SEM model. The results indicated that work stress was positively associated with emotional exhaustion, whereas emotional exhaustion was negatively associated with life satisfaction. Connectedness to nature and work engagement were involved in this indirect association: higher emotional exhaustion was associated with lower levels of connectedness to nature and work engagement, while higher levels of connectedness to nature and work engagement were associated with higher life satisfaction. The findings suggest that connectedness to nature and work engagement may be relevant psychological and work-related resources in this relationship.

## 1. Introduction

Work stress is the result of the characteristics of an environment that the worker perceives as threatening (Ezenwaji et al., 2019; M. Zhang et al., 2021), and may seriously compromise their health, satisfaction, capacity, and productivity (Ezenwaji et al., 2019). When such stress persists over long periods of time, such as under heavy workloads or in toxic workplaces (Kurniawan et al., 2024), it may be associated with emotional exhaustion and with symptoms of what is known as burnout syndrome (Ezenwaji et al., 2019; Marcionetti & Castelli, 2023; Maslach & Jackson, 1981) This syndrome, in turn, has been associated with lower workers’ life satisfaction and with symptoms such as anxiety, depression, post-traumatic stress disorder, low satisfaction, substance abuse, and an increase in suicide rates (Marcionetti & Castelli, 2023; Maslach & Jackson, 1981; Moscu et al., 2023; Raudenská et al., 2020). In response to these challenges, recent research has increasingly focused on identifying personal and contextual resources that may be associated with lower stress-related outcomes and better employee well-being (Gritzka et al., 2020; Landells & Albrecht, 2019).

Due to the negative consequences of work stress, over recent years, workplace interventions have been implemented to try and support workers’ health (Sadick & Kamardeen, 2020), and much research has been conducted to try and identify factors that may be associated with lower levels of stress-related strain. Work engagement has been associated with lower levels of stress-related outcomes and burnout symptoms and is therefore considered an important element in understanding well-being in work environments (Ruiz-Frutos et al., 2021; Yang et al., 2022). From this perspective, engagement is often conceptualised as an important psychological resource that has been linked to positive work-related outcomes and to employees’ resilience in the face of demanding work conditions (Bernales-Turpo et al., 2022; Foà et al., 2020).

Contact with nature has also been identified as a potential resource associated with higher life satisfaction, better attention and memory, and recovery from stress (Tomasi et al., 2020). The presence of natural elements in work environments has been associated with better recovery from symptoms linked to mental disorders and has also been found to be associated with creativity, sociability, engagement, and work satisfaction (Brossoit et al., 2025; Chang et al., 2020; Grahn et al., 2017). Emerging organisational research further suggests that nature experiences outside work may also be relevant for employee functioning. Evening nature contact has been linked to higher beginning-of-workday positive affect and subsequent work effort, particularly among employees with stronger nature connectedness (Brossoit et al., 2024; Klotz et al., 2023). Similarly, natural amenities at home have been indirectly associated with job engagement through time spent outdoors and enjoyment of outdoor experiences (Brossoit et al., 2024). In addition to physical exposure to natural environments, research has also highlighted the role of connectedness to nature, understood as an individual’s psychological sense of connection and emotional bond with the natural world, as a factor associated with enhanced well-being and psychological functioning (Baceviciene & Jankauskiene, 2022; Chang et al., 2020; C. Zhang et al., 2023).

This study examines the relationship between work stress and emotional exhaustion among employees and explores how emotional exhaustion relates to life satisfaction. Given that the depletion of emotional resources constitutes a central phenomenon in occupational health research, understanding its associations with psychological functioning and work performance is fundamental (Foà et al., 2020; Raudenská et al., 2020). Understanding these processes may inform the design of strategies aimed at supporting employees’ physical and psychological well-being and addressing distress in work contexts.

Although similar associations have been studied, the joint role of dispositional connectedness to nature and work engagement in the association between emotional exhaustion and life satisfaction has received limited empirical attention (Klotz et al., 2023; C. Zhang et al., 2023). By addressing this gap, the present study seeks to advance the understanding of how diverse psychological and motivational resources may be involved in the relationship between stress and well-being processes.

In doing so, this research integrates perspectives from occupational health psychology and environmental psychology to explore how connectedness to nature and work engagement may be involved as psychological and work-related resources in the relationship between emotional exhaustion and life satisfaction. Specifically, the proposed model examines whether work stress is positively associated with emotional exhaustion and whether emotional exhaustion is negatively associated with life satisfaction, connectedness to nature, and work engagement. In addition, the model examines whether connectedness to nature and work engagement are positively associated with life satisfaction and whether these variables are involved in the indirect association between emotional exhaustion and life satisfaction.

### 1.1. Work Stress and Emotional Exhaustion

Work stress is associated with poorer physical and mental health, with implications for both employees and the organisation (Menardo et al., 2022; Muntean et al., 2022; Sadick & Kamardeen, 2020). The principal causes of this type of stress include high work demands, low control over the task at hand, poor role delimitation, poor internal change management, insufficient support from superiors and colleagues, and difficult interpersonal relationships (Menardo et al., 2022). These situations have been associated with poorer workers’ health, performance, productivity, and work satisfaction (Ezenwaji et al., 2019; Kurniawan et al., 2024; Menardo et al., 2022; Zborowska et al., 2021). Jobs require individuals to use cognitive resources such as creative thinking, problem-solving, and work-related skills. A non-optimal, unhealthy work environment may be associated with lower performance, involvement, and engagement, and with higher absenteeism and staff turnover, thereby reducing organisational productivity (Menardo et al., 2022; Sadick & Kamardeen, 2020; Zborowska et al., 2021).

In sum, work stress has multiple causes and has been linked to several outcomes for both workers and organisations. Inadequate coping mechanisms and unfavourable psychosocial factors also contribute to physical and emotional exhaustion, generating feelings of work-related failure and frustration (Ezenwaji et al., 2019; Raudenská et al., 2020). If not dealt with properly, these factors may be associated with burnout, a syndrome that can seriously compromise workers’ health and well-being (Foà et al., 2020; Maslach & Jackson, 1981; Maslach et al., 2001; Sadick & Kamardeen, 2020). Burnout has been associated with poorer physical and psychological health, as well as difficulties in interpersonal relationships. Those suffering from this syndrome may manifest symptoms such as lack of engagement, absenteeism, and a drop in work-related performance and productivity (Bernales-Turpo et al., 2022; Marcionetti & Castelli, 2023).

Maslach’s model describes burnout as being composed of three sub-dimensions: Depersonalisation refers to a mental distancing from one’s job, impersonal behaviours, and negative responses to others. Reduced personal efficacy refers to a decrease in feelings of success and work-related competence. Finally, emotional exhaustion, which is often considered the core component of the syndrome and the most widely studied dimension (Maslach et al., 2001), is characterised by feelings of work-related overload and fatigue and may prompt individuals to disconnect emotionally and cognitively from their job as a strategy for coping with work overload (Foà et al., 2020; Maslach & Jackson, 1981; Maslach et al., 2001; Moscu et al., 2023; Raudenská et al., 2020; Yang et al., 2022; Zborowska et al., 2021).

Most studies to date have focused on the education and health professions, observing a negative association between stress and the health of employees working in these fields (Foà et al., 2020; Marcionetti & Castelli, 2023; Moscu et al., 2023; Zborowska et al., 2021). Emotional exhaustion, which manifests as a negative, prolonged response to stressors, is a particularly serious condition in this context, since it is negatively associated with workers’ mood, health, and well-being (Kurniawan et al., 2024; Zborowska et al., 2021). Consequently, when analysing engagement and Job Demands-Resources theory, we will focus particularly on the close relationship that exists between stress and emotional exhaustion.

**Figure 1:**
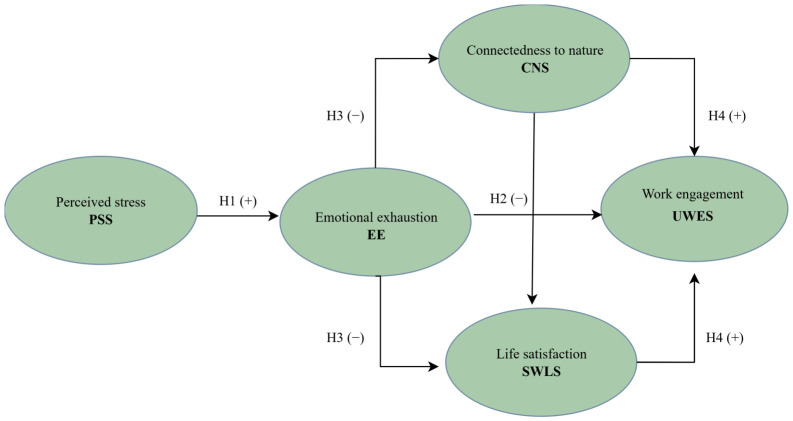
Hypothesised model of the relationships among perceived stress, emotional exhaustion, connectedness to nature, work engagement, and life satisfaction. Note. PSS = Perceived Stress Scale; EE = Emotional Exhaustion, assessed using the Emotional Exhaustion subscale of the Maslach Burnout Inventory–Human Services Survey; CNS = Connectedness to Nature Scale; UWES = Utrecht Work Engagement Scale; SWLS = Satisfaction with Life Scale. H5 denotes the serial indirect pathway from emotional exhaustion to life satisfaction through connectedness to nature and work engagement: EE → CNS → UWES → SWLS.

In light of the above, the following hypothesis is proposed (Figure 1):

**H1.**  *Work stress will be positively associated with emotional exhaustion.*

### 1.2. Life Satisfaction

Employees’ experience at work is associated with external factors, such as their health, financial situation, social relationships, and free time. Understanding these contextual factors is necessary in order to obtain a comprehensive view of employees’ well-being (Luan et al., 2022; C. Zhang et al., 2023). In this sense, overall life satisfaction may also be associated with job choice and work performance (Ampofo, 2021; Bernales-Turpo et al., 2022; Marcionetti & Castelli, 2023; Muntean et al., 2022).

Life satisfaction is an individual’s subjective assessment of their life in general, considering both positive and negative aspects (Demerouti et al., 2005; Luan et al., 2022; Merino et al., 2021; Muntean et al., 2022). It is closely linked to achieving positive outcomes in the personal, psychological, and social domains, and in turn is positively associated with those domains. Moreover, it is closely connected to family, leisure, occupation, and income (Ampofo, 2021; Marcionetti & Castelli, 2023; C. Zhang et al., 2023), since it is related to social conditions and personal achievements, and manifests itself in different social, affective, behavioural, and work-related contexts (Merino et al., 2021).

Spillover theory (Demerouti et al., 2005; Luan et al., 2022) postulates a link between an individual’s life and work, arguing that their experience in one area may be related to the other. From a theoretical and empirical perspective, this theory supports a relationship between life satisfaction and job satisfaction since it holds that an individual’s experience in one role may be associated with their experience in another role through a kind of intra-individual transmission of stress or tension from one domain to another. According to this theory, people who live a happy life also tend to feel more satisfied with their job, and vice versa (Demerouti et al., 2005; Luan et al., 2022; Moscu et al., 2023).

Life satisfaction may also be lower among employees reporting higher emotional exhaustion, as this negative state is linked to poorer work performance, lower perceived task control, and difficulties in everyday interactions, all of which may be related to lower overall quality of life (Kurniawan et al., 2024; Luan et al., 2022; Marcionetti & Castelli, 2023; Maslach et al., 2001; Moscu et al., 2023; Zborowska et al., 2021).

In light of the empirical evidence outlined above, the following hypothesis is proposed (Figure 1):

**H2.**  *Emotional exhaustion will be negatively associated with life satisfaction*.

### 1.3. Work Engagement

Work engagement is defined as a positive, fulfilling mental state associated with work. It is different from work addiction in that, although engaged employees enjoy their job, they know how to maintain a good balance between their work life and their personal life (W. Schaufeli, 2021). Engagement has often been conceptualised as distinct from burnout and as reflecting a healthy approach to work. It is made up of three dimensions: vigour, characterised by resilience, high levels of mental energy, enthusiasm, and appropriate strategies for coping with difficult situations; dedication, driven by effort, inspiration, and work-related challenges; and absorption, or the ability to concentrate without getting distracted (Ampofo, 2021; Foà et al., 2020; W. Schaufeli, 2021; Yang et al., 2022; M. Zhang et al., 2021). Highly engaged workers tend to demonstrate effective performance, optimism, and perseverance, which are associated with higher levels of job satisfaction and well-being (Brossoit et al., 2024; Foà et al., 2020; Hui & Aye, 2018; Ruiz-Frutos et al., 2021). The relationship between work engagement and life satisfaction, however, has been less widely explored (Ampofo, 2021).

Job Demands-Resources theory focuses on the positive and negative factors that may be generated at work (Bakker & Demerouti, 2007, 2013). The theory suggests that engagement is related to the interaction between one’s job, one’s personal resources, such as self-efficacy, and individual and organisational achievements, such as individual satisfaction and performance (Foà et al., 2020; Landells & Albrecht, 2019). Job demands, such as manipulation, abuse of authority, or gossip among colleagues, are associated with the depletion of employees’ energy and with higher stress. They are among the main factors associated with emotional exhaustion and health issues among workers and have been linked to engagement and job satisfaction (Bakker & Demerouti, 2007, 2013; Bernales-Turpo et al., 2022; Kurniawan et al., 2024; Landells & Albrecht, 2019; Marcionetti & Castelli, 2023; W. Schaufeli, 2021; Yang et al., 2022). Job resources, on the other hand, which include elements such as collaboration between colleagues and a good work climate, are associated with higher engagement and may be linked to lower emotional exhaustion, as they may encourage employees to view their job as a challenge rather than as a source of stress (Bakker & Demerouti, 2007; Bernales-Turpo et al., 2022; Foà et al., 2020; Landells & Albrecht, 2019; Moscu et al., 2023; Ruiz-Frutos et al., 2021). However, the relationship between work engagement and life satisfaction has received comparatively less empirical attention.

Previous research conducted with health professionals has suggested that work engagement may be involved in the indirect associations between burnout, life satisfaction, and job performance, and may help explain how burnout relates to work-related outcomes and job satisfaction (Bernales-Turpo et al., 2022; Foà et al., 2020).

### 1.4. Connectedness to Nature

Feeling connected to nature has been associated with benefits for human health, life satisfaction, and physical and psychological well-being (C. Zhang et al., 2023). Recent studies have reported that enjoying the natural landscape and contemplating one’s environment through a window may contribute to these benefits (Baceviciene & Jankauskiene, 2022; Brossoit et al., 2025; Chang et al., 2020; Sadick & Kamardeen, 2020; Tomasi et al., 2020; Yu et al., 2020). However, the design of work environments rarely takes into account the importance of ensuring the presence of natural elements such as plants, pictures of landscapes, windows that provide natural light, or outside spaces in which workers can rest and socialise with each other (Menardo et al., 2022; Yu et al., 2020).

Roger Ulrich’s Stress Recovery Theory (Ulrich, 1983) proposes that humans’ biological predisposition (Gritzka et al., 2020; Tomasi et al., 2020) to natural environments may help reduce stress (Menardo et al., 2022; Sadick & Kamardeen, 2020). Specifically, the theory suggests that contemplating natural environments may be associated with a sense of connection between the individual and nature, as well as with indicators of psychological restoration, such as higher positive emotions, lower emotional and physical fatigue, and better cognitive functioning (Baceviciene & Jankauskiene, 2022; Klotz et al., 2023; Mihandoust et al., 2021; Yu et al., 2020). The effect may be twofold: (a) physical recovery; which may involve releasing muscle tension and reducing heart rate and blood pressure; and (b) psychological recovery; fostering and stimulating positive affective states and moods (Hähn et al., 2021; Sadick & Kamardeen, 2020; Tomasi et al., 2020).

Other authors have found that proximity to nature may be associated with improved quality of life, a relationship that may be mediated by nature exposure and connectedness to nature (Baceviciene & Jankauskiene, 2022). Both direct exposure to real natural environments and the presence of artificial elements have been associated with lower anger, better stress management, and lower emotional exhaustion at work, and may therefore be related to better health and higher feelings of happiness (Brossoit et al., 2025). For example, when workers looked after plants in their offices, they were observed to experience an increase in personal satisfaction and a reduction in work-related stress (Hähn et al., 2021; Menardo et al., 2022; Mihandoust et al., 2021; Sadick & Kamardeen, 2020). Similarly, previous research in healthcare settings has suggested that employees with views of nature and access to hospital gardens during breaks may report fewer symptoms of emotional exhaustion (Mihandoust et al., 2021). In addition, being able to look out at nature through a window has been associated with higher life satisfaction (Chang et al., 2020). Furthermore, findings regarding the quantity and distance of plants in relation to workers support the idea that having plants indoors is associated with lower stress (Menardo et al., 2022).

In light of the above, this study explores whether emotional exhaustion is associated with lower levels of connectedness to nature, understood as a weaker subjective sense of connection and emotional bond with the natural environment, and how this association may relate to work engagement. In the present study, connectedness to nature is examined as a subjective psychological construct, rather than as objective exposure to workplace natural elements such as plants, natural light, green spaces, or views of nature.

The following hypothesis is therefore formulated (Figure 1):

**H3.**  *Emotional exhaustion will be negatively associated with connectedness to nature and work engagement.*

### 1.5. The Influence of Connectedness to Nature and Work Engagement

Previous research has suggested that contemplating natural environments may be associated with disconnection from work, lower stress, and better recovery of work capacity and physical and mental health (Gritzka et al., 2020; Menardo et al., 2022). Work engagement has also been shown to be involved in the association between burnout and job performance (Bernales-Turpo et al., 2022), as well as to correlate negatively with emotional exhaustion and positively with job satisfaction (Gritzka et al., 2020; Menardo et al., 2022; Moscu et al., 2023).

To optimise engagement, it is important to disconnect and recover from the fatigue generated by the workload (Ampofo, 2021), a process that connectedness to nature may be associated with (Baceviciene & Jankauskiene, 2022; Hähn et al., 2021) by fostering an affinity between nature and humans, which may be related to higher levels of engagement (Klotz et al., 2023). Consequently, connectedness to nature in work contexts may be relevant to workers’ positive emotions, personal well-being, and organisational outcomes (Baceviciene & Jankauskiene, 2022; Bernales-Turpo et al., 2022; Brossoit et al., 2025; Hähn et al., 2021). It may also be associated with a more collaborative work environment and higher life satisfaction (Baceviciene & Jankauskiene, 2022; Bernales-Turpo et al., 2022; Brossoit et al., 2025; Hähn et al., 2021). It is important to distinguish this dispositional trait from actual workplace nature exposure; while connectedness to nature is a psychological construct, previous research on the physical inclusion of natural elements in workplaces has also suggested that such elements may be associated with a relaxing atmosphere, better concentration, cognitive functioning, and worker productivity (Brossoit et al., 2024; Hähn et al., 2021; Yu et al., 2020).

As a result, connectedness to nature and work engagement may be relevant psychological and work-related resources for understanding workers’ health and well-being. Although similar associations have been studied, the combined role of connectedness to nature and work engagement in the associations between emotional exhaustion and life satisfaction among workers has received relatively limited attention.

The following hypotheses are therefore formulated (Figure 1):

**H4.**  *Connectedness to nature and work engagement will be positively associated with life satisfaction.*

**H5.**  *Connectedness to nature and work engagement will be involved in the indirect association between emotional exhaustion and life satisfaction.*

## 2. Materials and Methods

### 2.1. Participants

Participants were 1851 active Spanish workers (43.5% men and 56.5% women), aged between 19 and 83 years (*M* = 39.34; *SD* = 11.0). The sample was occupationally diverse: participants were mainly technical and support professionals (33.2%), administrative employees (20.4%), middle managers (18.4%), other occupational groups (10.3%), civil servants and healthcare professionals (9.8%), and unskilled workers (7.9%). One participant did not disclose this information. In terms of education level, most participants reported having higher education qualifications (Bachelor’s, Master’s, Engineering degree) (60.3%). The remaining 39.7% were distributed among Vocational Training (19.9%), High School (11.9%), Primary Education (7.8%), and no formal Education (0.1%). Overall, participants reported having worked for the same company or organisation for an average of 10.2 years (*SD* = 10.1). Moreover, 85.4% of participants reported having a full-time job, and 14.6% reported working on a part-time or hourly basis.

### 2.2. Procedure

The study was approved by the UNED Research Ethics Committee (BICI, 24 April 2018; CG modification, 30 April 2019; approval certificate received on 9 September 2019; reference number PREXT-9-9-19). Data collection was conducted through a combination of direct recruitment by the research team and collaboration with undergraduate Psychology students at the National Distance Education University (UNED), who acted as research assistants. Specifically, students were invited to disseminate the survey among individuals within their personal networks who were currently employed, with each student asked to recruit up to ten participants. Students did not complete the questionnaire themselves. Although student-recruited samples have been subject to methodological debate, prior research suggests that they can provide data of comparable quality to more traditional recruitment strategies when appropriate procedures are followed (Demerouti & Rispens, 2014).

Participants were contacted through various communication channels, including email and social media platforms. As recruitment relied on voluntary participation and network-based dissemination, the exact number of individuals who received the questionnaire could not be determined. Data were collected between January and October 2019 through an online survey. A total of 1857 questionnaires were completed. After excluding six respondents who reported being unemployed at the time of the survey, the final analytical sample consisted of 1851 participants.

To minimise missing data and ensure response completeness, all questionnaire items were set as mandatory. Consequently, only fully completed questionnaires were included in the analyses. Prior to participation, respondents were informed about the aims of the study, the voluntary nature of their participation, and the confidentiality of their responses, and they provided informed consent. The questionnaire required approximately 15–20 min to complete.

### 2.3. Instruments

Participants completed the scales outlined below.

The Perceived Stress Scale (PSS) (Cohen et al., 1983; adapted to Spanish by Remor, 2006) was used to measure stress. This instrument, which comprises 14 items, measures a person’s sense of being in control of life situations and whether or not they feel overwhelmed by them (e.g., “In the past month, how often have you been affected by something that happened unexpectedly?”). The response scale ranges from 0 (Never) to 4 (Very often). Cronbach’s alpha was 0.87, indicating good internal consistency.

To measure emotional exhaustion, the Emotional Exhaustion subscale of the Maslach Burnout Inventory (MBI-HSS) (Maslach et al., 1996; Spanish version by Gil-Monte, 2005) was used. This dimension (five items) measures professional burnout, or in other words, the emotional exhaustion related to work demands (e.g., “I am emotionally exhausted by my job”). The response scale ranges from 0 (Never) to 6 (Every day). Cronbach’s alpha was 0.92, indicating good internal consistency.

The Satisfaction with Life Scale (SWLS) (Diener et al., 1985; Spanish version by Atienza et al., 2003) was used to measure life satisfaction. The scale comprises five items and measures subjective well-being by focusing on overall life satisfaction. Life satisfaction is gauged through the cognitive judgment that a person makes about their life. The different items that make up the scale enable respondents to generally assess what they have achieved in life and their degree of satisfaction. High scores on the SWLS indicate greater satisfaction with life (e.g., “The type of life I lead is similar to the one I always dreamed of”). The response scale ranges from 1 (Strongly disagree) to 5 (Strongly agree). Cronbach’s alpha was 0.87, indicating good internal consistency.

The Utrecht Work Engagement Scale (UWES-9) (W. B. Schaufeli & Bakker, 2003; Spanish version by Domínguez-Salas et al., 2022) was used to measure work engagement. This scale, which consists of nine items, measures how people manage their emotions and channel physical and cognitive energy into work through three dimensions: Vigour (three items) assesses levels of energy and resilience, i.e., whether tasks are performed with enthusiasm, resulting in less resistance to their completion (e.g., “I am enthusiastic about my job”); Dedication (three items) assesses the respondent’s feeling of enthusiasm and pride in the work done, i.e., whether they identify with their work and whether they feel it is inspiring and challenging (e.g., “I am proud of my work”); and Absorption (three items) refers to the degree to which the respondent is happily immersed in their work (e.g., “I get ‘carried away’ by my work”). Higher UWES scores indicate greater work engagement. The response scale ranges from 0 (Never) to 6 (Every day). Cronbach’s alpha for the total scale was 0.92, whereas for the vigour dimension it was 0.87 and for both dedication and absorption it was 0.81. All values indicate good internal consistency.

Finally, the Connectedness to Nature Scale (CNS) (Mayer & Frantz, 2004) was used to measure subjective connectedness to nature. To ensure idiomatic equivalence between the original English version of the questionnaire and the Spanish version, a blind back-translation procedure was followed (Beaton et al., 2000; Bracken & Barona, 1991). First, the research team translated the original questionnaire from English into Spanish. Next, a bilingual person with no knowledge of the original version back-translated the Spanish instrument into English. Finally, the research team confirmed the equivalence between the items in the original version and the back-translated English version. The scale (14 items) reflects the degree to which respondents feel emotionally and cognitively connected to the natural world. Therefore, this instrument allowed us to assess connectedness as a subjective psychological trait rather than objective environmental exposure. Higher CNS scores indicate a deeper connection with nature (e.g., “I often feel a sense of oneness with the natural world around me”). The response scale ranges from 1 (Strongly disagree) to 5 (Strongly agree). The Cronbach’s alpha was 0.87, indicating good internal consistency.

### 2.4. Data Analysis

First, a descriptive analysis was performed using IBM SPSS Statistics version 27.0 software to explore the relationships between stress, emotional exhaustion, life satisfaction, work engagement, and connectedness to nature. Prior to the main analyses, the data were screened for distributional assumptions, missing values, and potential outliers. Univariate outliers were inspected using boxplots and extreme value tables, and residual diagnostics were examined using standardised residuals and residual scatterplots. In the regression model, standardised residuals ranged from −3.596 to 3.801. Although some observations exceeded the conventional ±3 threshold, these cases were retained because they did not indicate systematic data-entry errors or distort the overall pattern of results. Thus, no additional cases were removed due to outliers. Next, Structural Equation Modelling (SEM) was performed using IBM AMOS version 27.0 (Arbuckle, 2006). For the SEM analyses, AMOS normality diagnostics and Mahalanobis distance were examined to assess multivariate normality and potential multivariate outliers. No cases were removed on the basis of outlier diagnostics. The final SPSS analytical sample consisted of 1851 participants, whereas the SEM analyses were conducted with 1850 valid cases.

Maximum likelihood estimation methods were used, and the input for each analysis was the covariance matrix of the variables. As recommended by Hoyle and Panter (1995), several different fit indexes were calculated to provide a better assessment of the models: chi-square/degrees of freedom ratio (χ^2^/df), root mean square error of approximation (RMSEA), comparative fit index (CFI), Tucker–Lewis index (TLI), incremental fit index (IFI), and normed fit index (NFI). RMSEA criteria for fit are as follows: <0.05 (good fit), 0.05 to 0.08 (satisfactory fit), 0.08 to 0.10 (mediocre fit), and >0.10 (unacceptable fit). CFI, TLI, IFI, and NFI values range between 0 and 1, with values between 0.90 and 0.95 indicating acceptable model fit and values >0.95 indicating good model fit. No sociodemographic variables were entered as statistical control variables in the final SEM model; therefore, the results are reported as unadjusted structural associations.

To test the mediation hypotheses, the multi-step SEM approach proposed by Holmbeck (1997) was applied. The analysis was conducted in three steps. First, a direct-effects model was estimated, including a pathway from stress to emotional exhaustion and a direct pathway from emotional exhaustion (predictor) to life satisfaction (outcome). Second, a full mediation model was tested by specifying only the indirect pathways between emotional exhaustion and life satisfaction through connectedness to nature and work engagement (mediators). Finally, a partial mediation model was estimated, including both the direct pathway between emotional exhaustion and life satisfaction and the indirect pathways through the proposed mediators. In all models, stress was retained as an antecedent variable predicting emotional exhaustion.

Indirect effects were estimated using 5000 bootstrap samples and 95% bias-corrected confidence intervals. Standardised indirect effects were reported for consistency with the direct and total effects. Indirect effects were considered statistically significant when the confidence interval did not include zero.

Full mediation was considered to occur when (a) the partial mediation model did not show a significantly better fit than the full mediation model and (b) the direct effect between emotional exhaustion and life satisfaction became non-significant after including the mediating variables in the model. Partial mediation was inferred when the indirect effect was significant and the direct effect remained statistically significant.

## 3. Results

The results of the descriptive and correlational analyses are presented here. The means and standard deviations are also shown, along with the correlations between the variables. Table 1 indicates significant relationships between the variables. Stress and emotional exhaustion were negatively associated with life satisfaction, connectedness to nature, and engagement. Furthermore, a positive association was found between stress and emotional exhaustion. As expected, life satisfaction correlated positively with connectedness to nature and engagement.

The final SEM model showed an acceptable fit to the data, as shown in Table 2.

**Figure 2:**
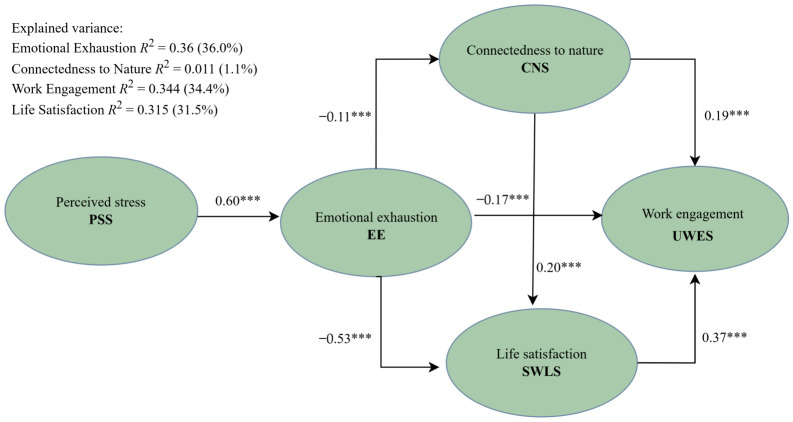
Multiple mediation model. Note. PSS = Perceived Stress Scale; EE = Emotional Exhaustion, assessed using the Emotional Exhaustion subscale of the Maslach Burnout Inventory–Human Services Survey; CNS = Connectedness to Nature Scale; UWES = Utrecht Work Engagement Scale; SWLS = Satisfaction with Life Scale. Standardised path coefficients are shown; *R*^2^ values indicate explained variance. H5 denotes the serial indirect pathway EE → CNS → UWES → SWLS. *** *p* < 0.001.

The results of the tested model are shown in Figure 2. Perceived stress was positively associated with emotional exhaustion (*β* = 0.60, *p* < 0.001), which was consistent with Hypothesis 1. Emotional exhaustion was negatively associated with life satisfaction (*β* = −0.17, *p* < 0.001), which was consistent with Hypothesis 2. Emotional exhaustion was also negatively associated with connectedness to nature (*β* = −0.11, *p* < 0.001) and work engagement (*β* = −0.53, *p* < 0.001), which was consistent with Hypothesis 3. In addition, connectedness to nature was positively associated with work engagement (*β* = 0.20, *p* < 0.001), in line with the hypothesised serial pathway. Connectedness to nature (*β* = 0.19, *p* < 0.001) and work engagement (*β* = 0.37, *p* < 0.001) were positively associated with life satisfaction, which was consistent with Hypothesis 4. Finally, the hypothesised serial indirect association between emotional exhaustion and life satisfaction through connectedness to nature and work engagement was statistically significant, which was consistent with Hypothesis 5. The specific serial indirect effect, estimated in unstandardised terms, was significant (*b* = −0.004, *SE* = 0.001, 95% BC bootstrap CI [−0.008, −0.002], *p* < 0.001). The standardised total indirect effect of emotional exhaustion on life satisfaction through the mediators was also statistically significant (*β* = −0.224, *SE* = 0.019, 95% CI [−0.262, −0.189], *p* < 0.001, *PM* = 0.569). Because the serial indirect effect and the total indirect effect are reported using different metrics and refer to different effects, these coefficients should not be directly compared. A breakdown of the unstandardised and standardised direct, specific indirect, serial indirect, total indirect, and total effects is presented in Table 3. Overall, the indirect pathways accounted for approximately 56.9% of the total association between emotional exhaustion and life satisfaction.

## 4. Discussion

The aim of the present study was to analyse the relationship between stress and emotional exhaustion and determine how emotional exhaustion is associated with life satisfaction. We also expected connectedness to nature and work engagement to be involved in the relationship between emotional exhaustion and life satisfaction among workers.

The results revealed that perceived stress was indeed significantly associated with the symptoms of emotional exhaustion, thereby confirming our first hypothesis. Stressful situations that are sustained over time, combined with psychosocial factors such as job insecurity, poor social support, or excessive workload, may contribute to a situation of emotional exhaustion that is highly relevant to workers’ health and well-being. These findings are consistent with those reported by previous studies (Ezenwaji et al., 2019; Kurniawan et al., 2024; Maslach et al., 2001; Moscu et al., 2023).

The results also confirmed our second hypothesis by demonstrating that, consistently with that observed in previous studies (Demerouti et al., 2005; Kurniawan et al., 2024; Marcionetti & Castelli, 2023; Moscu et al., 2023; Yu et al., 2020), emotional exhaustion is negatively associated with workers’ life satisfaction. The symptoms of emotional exhaustion, which include insomnia, anxiety, and depression, are related to high absenteeism and staff turnover, which in turn are negatively associated with job and life satisfaction. Indeed, it has been confirmed that, among employees working in the educational, nursing, and hospitality sectors, emotional exhaustion may limit daily performance and relate negatively to individuals’ work and life satisfaction (Ezenwaji et al., 2019; Marcionetti & Castelli, 2023; Yu et al., 2020). For their part, high job demands may be negatively related to both workers’ family life and their satisfaction. In other words, emotional exhaustion and life satisfaction may spill over into close relationships and the well-being of loved ones. Both positive and negative experiences may be relevant to not only individuals’ personal and work environments, but also their interpersonal relationships (Demerouti et al., 2005; Luan et al., 2022).

The negative association observed between emotional exhaustion and both connectedness to nature and work engagement supported our third hypothesis. Specifically, higher levels of emotional exhaustion were associated with lower levels of connectedness to nature, understood as a weaker subjective connection and emotional bond with the natural environment, and with lower levels of work engagement. While our study assessed connectedness to nature as a psychological construct rather than actual workplace nature exposure, our findings align with previous studies suggesting that the presence of natural elements in workspaces is associated with lower emotional exhaustion (Chang et al., 2020; Hähn et al., 2021; Kurniawan et al., 2024; Menardo et al., 2022; Sadick & Kamardeen, 2020). Similarly, other studies support our proposal that emotional exhaustion is also negatively related to engagement, and that strengthening interpersonal relations and fostering a collaborative atmosphere may be associated with higher engagement levels (Bakker & Demerouti, 2013; Bernales-Turpo et al., 2022; Foà et al., 2020; Moscu et al., 2023; Ruiz-Frutos et al., 2021; M. Zhang et al., 2021).

In support of our fourth hypothesis, the data revealed that both connectedness to nature and work engagement are positively associated with life satisfaction. This indicates that when workers feel connected to nature and are more engaged in their work, their level of life satisfaction is higher. This may be related to the restorative benefits of connectedness to nature on subjective health and well-being (Baceviciene & Jankauskiene, 2022; C. Zhang et al., 2023). These findings are consistent with those reported by other studies that found a strong positive link between life satisfaction and work engagement (Baceviciene & Jankauskiene, 2022; Bernales-Turpo et al., 2022).

Finally, our results were consistent with our fifth hypothesis. Work engagement and connectedness to nature statistically accounted for part of the association between emotional exhaustion and life satisfaction. Although both indirect pathways were statistically significant, the pathway involving work engagement was descriptively stronger in our model than the pathway involving connectedness to nature. This suggests that, although dispositional characteristics such as nature connectedness may be relevant to well-being, proximal work-related resources such as work engagement may represent a more prominent explanatory pathway in the association between emotional exhaustion and life satisfaction. In other words, both variables may help explain the negative association between emotional exhaustion and life satisfaction among workers (Brossoit et al., 2025; Chang et al., 2020; Foà et al., 2020; Moscu et al., 2023; Sadick & Kamardeen, 2020; Yang et al., 2022). Similar results have been found previously in relation to connectedness to nature mediating between proximity to nature and quality of life (Baceviciene & Jankauskiene, 2022; C. Zhang et al., 2023).

These same authors also suggested that stronger connectedness to nature is associated with better stress recovery, cognitive performance, and life satisfaction. Consequently, feeling connected to nature may be linked to higher life satisfaction (Chang et al., 2020; Sadick & Kamardeen, 2020; Tomasi et al., 2020; C. Zhang et al., 2023). In relation to work engagement, highly engaged workers tend to invest attention, energy, and interest in their work tasks (Foà et al., 2020; W. Schaufeli, 2021). In a study carried out with health professionals examining similar indirect relationships, work engagement was found to be involved in the association among burnout, life satisfaction, and job performance (Bernales-Turpo et al., 2022). In the present model, work engagement may be interpreted as a complementary work-related resource involved in the association between emotional exhaustion and life satisfaction. Specifically, within the hypothesised model, higher emotional exhaustion was associated with lower work engagement, which was also associated with lower life satisfaction.

Overall, these findings suggest that both connectedness to nature and work engagement may function as complementary resources in the relationship between emotional exhaustion and life satisfaction (Menardo et al., 2022). While connectedness to nature reflects individuals’ psychological relationship with natural environments, work engagement represents a motivational resource linked to work-related well-being (Baceviciene & Jankauskiene, 2022; Hui & Aye, 2018; Landells & Albrecht, 2019; Menardo et al., 2022). Considering both environmental and motivational resources within the same model may therefore help to better understand why some employees report higher life satisfaction in the context of emotional exhaustion.

### Limitations and Future Research

This study has certain limitations that should be acknowledged. First, its cross-sectional design precludes establishing causal relationships between the variables studied. Therefore, future research should employ longitudinal designs to examine the temporal ordering of the mediational pathways identified in our model. Second, although the proposed model highlights the mediating role of connectedness to nature, our study focused on this construct as a subjective psychological bond with the natural environment and did not consider how the physical characteristics of the work environment might relate to its development. In particular, we did not differentiate between potential sources of contact with nature in the workplace, such as access to outdoor natural spaces, the presence of indoor plants, or access to natural light.

Based on this limitation, future research could examine how this personal resource interacts with the actual physical work environment. Although some studies have investigated the relationship between connectedness to nature and nature-based interventions in work contexts, a better understanding of how these dynamics operate together is still needed (Gritzka et al., 2020; Hui & Aye, 2018; Sadick & Kamardeen, 2020; C. Zhang et al., 2023). In this regard, future research could examine potential person–environment interactions, analysing whether employees with a higher dispositional connectedness to nature respond differently to nature exposure in the workplace. For instance, it would be valuable to analyse whether employees with a high dispositional connectedness to nature are more vulnerable to emotional exhaustion in workspaces lacking natural elements or whether, conversely, this characteristic allows them to benefit to a greater extent from specific nature-based interventions compared to those with a weaker connection (Chang et al., 2020; Klotz et al., 2023). Likewise, given that connectedness to nature has been associated with benefits for individual well-being, future research could examine whether this psychological bond also translates into broader organisational advantages, such as the promotion of pro-environmental behaviours at work (Baceviciene & Jankauskiene, 2022; Salazar et al., 2021).

Third, sociodemographic variables were not included as statistical control variables in the final SEM model. Future studies could therefore examine whether the observed associations remain stable after controlling for variables such as gender, age, educational level, occupational category, and job tenure, or whether these variables moderate the relationships between emotional exhaustion, connectedness to nature, work engagement, and life satisfaction.

Finally, future research could further explore potential serial mediation processes between these constructs, particularly the pathway from emotional exhaustion to life satisfaction through connectedness to nature and work engagement. This approach would provide a more detailed understanding of how these complementary resources are interrelated and how they are associated with lower emotional exhaustion and higher life satisfaction.

## 5. Conclusions

The present study provides insight into the statistical relationship between stress and emotional exhaustion, and the subsequent association of the latter variable with life satisfaction among workers. The study also highlights the mediating roles of connectedness to nature and work engagement in the relationship between emotional exhaustion and life satisfaction. By integrating these variables within the same explanatory model, the findings contribute to the growing literature on personal and motivational resources associated with workers’ well-being. In light of these results, organisations could consider work-environment and well-being initiatives that take employees’ psychological connection to nature and work engagement into account. Such initiatives should be understood as potentially relevant avenues for supporting employee well-being, although their effects should be examined in future longitudinal or intervention studies. Overall, our findings suggest that psychological connectedness to nature, together with work engagement, may represent relevant personal and work-related resources involved in the association between emotional exhaustion and life satisfaction.

## Figures and Tables

**Table 1:** Descriptive statistics and correlations.

	Descriptive Statistics		Correlations			
	*M*	*SD*	PSS	EE	SWLS	CNS
PSS	1.74	0.63				
EE	2.73	1.47	0.50 **			
SWLS	3.57	0.80	−0.50 **	−0.33 **		
CNS	3.65	0.67	−0.17 **	−0.11 **	0.25 **	
UWES	3.65	1.15	−0.31 **	−0.45 **	0.46 **	0.23 **

Note: PSS = Perceived Stress Scale; EE = Emotional Exhaustion, assessed using the Emotional Exhaustion subscale of the Maslach Burnout Inventory–Human Services Survey; CNS = Connectedness to Nature Scale; UWES = Utrecht Work Engagement Scale; SWLS = Satisfaction with Life Scale. *N* = 1851. *M* = Mean; *SD* = Standard Deviation. ** *p* < 0.01.

**Table 2:** Fit indices for the final SEM model.

Fit Index	Obtained Value	Reference Criterion	Interpretation
χ^2^	5043.235	-	-
df	991	-	-
*p*	<0.001	-	-
χ^2^/df	5.09	≤5 acceptable fit	Close to acceptable
RMSEA	0.047	<0.05 good fit	Good
90% CI for RMSEA	[0.046, 0.048]	Values below 0.05 indicate good fit	Good
CFI	0.916	≥0.90 acceptable fit	Acceptable
TLI	0.908	≥0.90 acceptable fit	Acceptable
IFI	0.916	≥0.90 acceptable fit	Acceptable
NFI	0.898	≥0.90 acceptable fit	Slightly below threshold

Note: SEM = Structural Equation Modelling; χ^2^ = chi-square; df = degrees of freedom; RMSEA = Root Mean Square Error of Approximation; CI = confidence interval; CFI = Comparative Fit Index; TLI = Tucker–Lewis Index; IFI = Incremental Fit Index; NFI = Normed Fit Index. The final SEM model was estimated with 1850 valid cases.

**Table 3:** Unstandardised and standardised direct, indirect, and total effects of emotional exhaustion on life satisfaction.

Effect	Estimate (*b*)	*SE* for *b*	95% BC Bootstrap CI for *b*	Estimate (*β*)	*p*
Specific indirect effects					
EE → CNS → SWLS	−0.012	0.004	[−0.019, −0.005]	−0.020	<0.001
EE → UWES → SWLS	−0.113	0.011	[−0.136, −0.092]	−0.196	<0.001
Serial indirect effect					
EE → CNS → UWES → SWLS	−0.004	0.001	[−0.008, −0.002]	−0.008	<0.001
Total indirect effect	−0.129	0.012	[−0.153, −0.107]	−0.224	<0.001
Direct effect, EE → SWLS	−0.097	0.016	-	−0.169	<0.001
Total effect	−0.226	0.017	[−0.259, −0.193]	−0.394	<0.001

Note: EE = Emotional Exhaustion; CNS = Connectedness to Nature Scale; UWES = Utrecht Work Engagement Scale; SWLS = Satisfaction with Life Scale. Unstandardised estimates, standard errors, and bias-corrected bootstrap confidence intervals are reported for *b*. Standardised estimates are reported as *β* to facilitate interpretation and comparison with the structural paths shown in Figure 2. BC = bias-corrected.

## Data Availability

The datasets generated and/or analysed during the current study are not publicly available due to confidentiality restrictions but are available from the corresponding author on reasonable request.
